# Sho1p Connects Glycolysis to Ras1-cAMP Signaling and Is Required for Microcolony Formation in Candida albicans

**DOI:** 10.1128/mSphere.00366-20

**Published:** 2020-07-08

**Authors:** Rohitashw Kumar, Malabika Maulik, Ruvini U. Pathirana, Paul J. Cullen, Mira Edgerton

**Affiliations:** a Department of Oral Biology, School of Dental Medicine, University at Buffalo, Buffalo, New York, USA; b Department of Biological Sciences, College of Arts and Sciences, University at Buffalo, Buffalo, New York, USA; University of Texas Health Science Center

**Keywords:** *Candida albicans*, Ras1-cAMP, Sho1, glycolysis, microcolonies

## Abstract

Candida albicans is an opportunistic, dimorphic fungus that causes candidiasis in immunocompromised people. C. albicans forms specialized structures called microcolonies that are important for surface adhesion and virulence. Microcolonies form in response to specific environmental conditions and require glycolytic substrates for optimal growth. However, fungal signaling pathways involved in sensing and transmitting these environmental cues to induce microcolony formation have not been identified. Here, we show that the C. albicans Ras1-cAMP cascade is required for microcolony formation, while the Cek1-MAP kinase pathway is not required, and Hog1 represses microcolony formation. The membrane protein Sho1, known to regulate the Cek1 pathway in yeasts, was indispensable for C. albicans microcolony formation but regulated the Ras1-cAMP pathway instead, based upon diminished intracellular levels of cAMP and reduced expression of core microcolony genes, including *HWP1*, *PGA10*, and *ECE1*, in C. albicans *sho1*Δ cells. Based upon predicted physical interactions between Sho1 and the glycolytic enzymes Pfk1, Fba1, Pgk1, and Cdc19, we hypothesized that Sho1 regulates Ras1-cAMP by establishing cellular energy levels produced by glycolysis. Indeed, microcolony formation was restored in C. albicans *sho1*Δ cells by addition of exogenous intermediates of glycolysis, including downstream products of each predicted interacting enzyme (fructose 1,6 bisphosphate, glyceraldehyde phosphate, 3-phosphoglyceric acid, and pyruvate). Thus, C. albicans Sho1 is an upstream regulator of the Ras1-cAMP signaling pathway that connects glycolytic metabolism to the formation of pathogenic microcolonies.

**IMPORTANCE** C. albicans microcolonies form extensive hyphal structures that enhance surface adherence and penetrate underlying tissues to promote fungal infections. This study examined the environmental conditions that promote microcolony formation and how these signals are relayed, in order to disrupt signaling and reduce pathogenesis. We found that a membrane-localized protein, Sho1, is an upstream regulator of glycolysis and required for Ras1-cAMP signaling. Sho1 controlled the Ras1-dependent expression of core microcolony genes involved in adhesion and virulence. This new regulatory function for Sho1 linking glycolysis to microcolony formation reveals a novel role for this fungal membrane protein.

## Abstract (precis)

C. albicans microcolonies form extensive hyphal structures that enhance surface adherence and penetrate underlying tissues to promote fungal infections. This study examined the environmental conditions that promote microcolony formation and how these signals are relayed, in order to disrupt signaling and reduce pathogenesis. We found that a membrane-localized protein, Sho1, is an upstream regulator of glycolysis and required for Ras1-cAMP signaling. Sho1 controlled the Ras1-dependent expression of core microcolony genes involved in adhesion and virulence. This new regulatory function for Sho1 linking glycolysis to microcolony formation reveals a novel role for this fungal membrane protein.

## INTRODUCTION

Fungal pathogens are a worldwide threat to human health. Candida albicans is an opportunistic fungus and the causative agent of oropharyngeal candidiasis (OPC), although it is typically a harmless commensal resident of the oral cavity (1). OPC develops in a wide spectrum of immunocompromised people, including those with untreated HIV infection, diabetics, cancer patients with mucositis, and people using steroids (2). Virulence of this organism can be traced to several properties, particularly its ability to germinate from a yeast cell type into a morphologically distinct hyphal (or filamentous) cell type (3).

Microcolonies are a type of filamentous growth that form more organized structures and are inherently more invasive than classical filamentous or biofilm growth due to coordinated expression of a set of virulence genes (4). Morphologically, microcolonies form a dense radiating network of hyphae originating from a single mother cell (4). *In vitro*, these structures were described previously as “embedded growth” that produces long hyphae that penetrate into the agar substrate (5). Neither classical filamentation nor biofilm regulatory pathways entirely account for microcolony development. Instead, we found that microcolony morphogenesis is controlled by the transcription factors Sfl1 and Sfl2 cooperating with at least three regulators of both filamentous and static biofilm growth (Rob1, Ndt80, and Efg1) (4). *Candida* species that produce hyphae and other filament-forming fungi, including Aspergillus fumigatus, form hyphal microcolonies (6). C. albicans mucosal infections *in vivo* appear as surface-localized plaques of fungal cells with extensive filamentation that closely resemble microcolonies *in vitro* (7). Hyphal microcolonies invade the underlying superficial epithelium to form dense interlocking filaments that help resist extraction by mechanical forces and form the “roots” of a mature biofilm.

C. albicans microcolonies form on both abiotic and epithelial surfaces at 37°C and 5% CO_2_, physiological conditions under which C. albicans causes systemic as well as superficial infections in the human host. Upon microcolony formation, cells selectively express a set of virulence genes, including *HWP1* and *HYR1* (encoding adhesion and/or invasion proteins), *ECE1* (encoding candidalysin, which permeabilizes host cells), and *PGA10* (involved in iron uptake) (4). However, classical filamentation pathways do not entirely account for microcolony development. We found that morphogenesis is controlled by two transcription factors (Sfl1 and Sfl2) cooperating with at least two important regulators of filamentous growth (Efg1 and Ndt80) (4, 7). In the budding yeast Saccharomyces cerevisiae, which offers a genetically amenable model for studying filamentous growth, Sfl1p (suppressor gene for flocculation) is a target of the cAMP-protein kinase A (PKA) pathway (8). In C. albicans, Sfl2 integrates the tricarboxylic acid (TCA) cycle and the Ras1-cAMP signaling pathway (9) to regulate hyphal growth. However, it is not known whether these pathways also regulate C. albicans microcolony formation.

**FIG 1:**
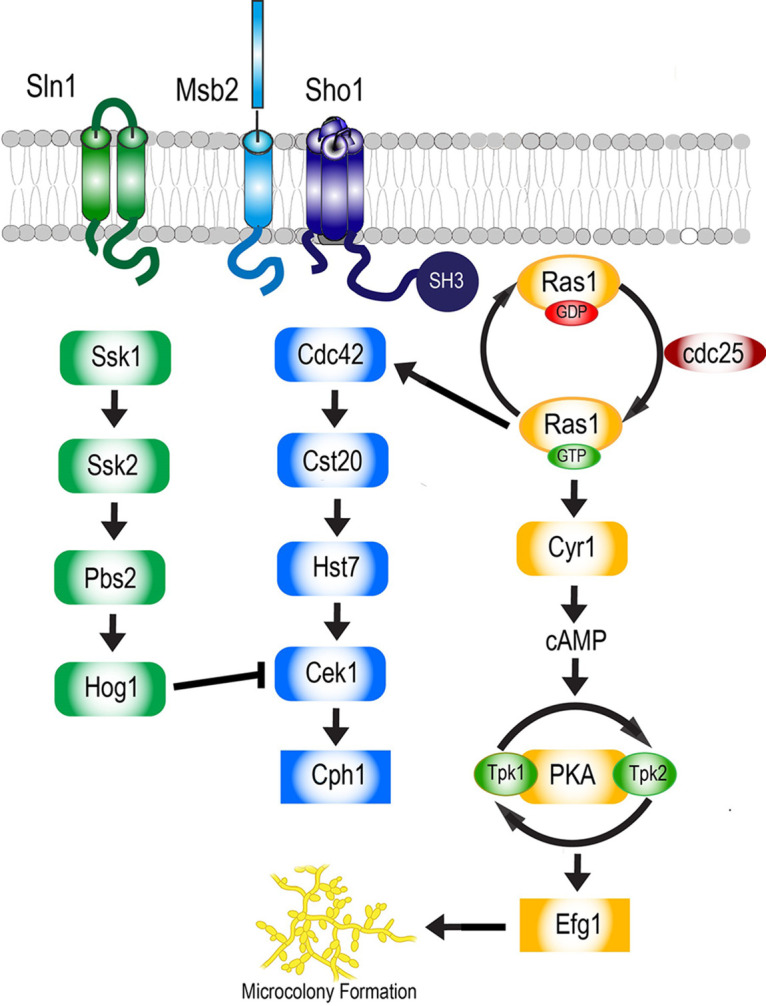
Signal transduction pathways that regulate C. albicans morphogenesis. C. albicans regulates filamentation by three main signaling pathways, which include the HOG MAPK pathway, the Cek1-mediated MAPK pathway, and the Ras1-cAMP pathway. Gene deletion mutants of each component of these MAP kinase pathways were examined for their ability to form filamentous microcolonies in RPMI 1640 at 37°C and 5% CO_2_.

Mitogen-activated protein kinase (MAP kinase) signaling cascades were first identified in S. cerevisiae and are highly conserved among fungi (10), as they transmit information about extracellular conditions to enable fungal cellular responses. Three major MAP kinase pathways (Hog1, Cek1, and Ras1-cAMP) have been well described in both C. albicans (Fig. 1) and S. cerevisiae. In both yeasts, the high osmolarity glycerol (HOG) pathway counters extracellular osmostress by Hog1 phosphorylation, which induces glycerol production as well as other adaptive responses, including ion transport, metabolism, and cell cycle progression (11). The C. albicans HOG1 pathway provides critical environmental adaptive mechanisms, such that *HOG1* deletion mutants are completely unable to colonize the gut (12). In S. cerevisiae, the Hog1 pathway coordinates adaptation to osmostress by two branches, the Sln1 branch and the Sho1/Msb2 branch, that converge to regulate Hog1 (13). However, in C. albicans, the MAP kinase Hog1 is induced by osmotic stress only through its membrane sensor, Sln1, which results in transcription of osmotic stress and other core stress genes (14, 15). C. albicans Hog1 signaling communicates with other filamentation pathways and acts as a repressor of the yeast-to-hypha switch, in part by functionally repressing Cek1 (16).

In both yeasts, Cek1 responds to fungal cell wall glycostructure damage that is sensed by a transmembrane mucin-like protein called Msb2 (17, 18). However, the C. albicans Cek1 MAP kinase (MAPK) pathway also controls hyphal differentiation and virulence (19), so that its loss results in defects in both invasive hyphal growth and biofilm formation (17). In C. albicans, Msb2 associates with the adaptor four-pass transmembrane protein Sho1 (14) to regulate downstream kinases (Fig. 1) that are dependent upon the presence of Sho1 (20). However, C. albicans Sho1 may also act in parallel with Msb2 in sensing cell wall damage caused by antifungal drugs, antimicrobial peptides, or temperature stress (12, 14, 16, 17, 21, 22).

S. cerevisiae Ras2 regulates nutrient signaling (23) and glucose activation of cAMP synthesis (24). However, in C. albicans, the Ras1-cAMP pathway plays a broader role in the response to a range of environmental stimuli, including temperature, serum, and pH, to induce filamentation (25, 26). In C. albicans, Ras1 is a small GTPase that exists in the cell in a GDP-bound inactive form (27). Upon activation by the guanine nucleotide exchange protein Cdc25, Ras1 binds to GTP and then activates its downstream target Cyr1 (Efg1 pathway) (26, 28). Cyr1 functions as an adenylate cyclase that regulates the production of cyclic AMP (cAMP) (29). In this capacity, Cyr1 plays a pivotal role in environmental sensing and functions in the derepression of protein kinase A (PKA) subunits, which are the proteins Tpk1 and Tpk2 in C. albicans. Tpk1 and Tpk2 in turn activate the transcription factor Efg1, which controls the expression of hypha-specific genes (30). Although these conserved Ras1-cAMP pathway components are known in both yeasts, most upstream sensors in C. albicans that link environmental cues with signaling to produce filamentation are not well understood.

Glycolysis is a central metabolic pathway that is essential for carbon utilization and energy production (31) but also is crucial for Ras1 activation in both S. cerevisiae and C. albicans (24, 32). Glucose is broken down into several intermediates before forming pyruvate (33), which then enters the tricarboxylic acid (TCA) cycle, ultimately contributing to mitochondrial respiration to produce ATP and CO_2_ (26). The energy status of the cell is the most important signal triggering C. albicans filamentation; thus, Ras1 signaling is highly dependent upon carbon metabolism, the TCA cycle, and ATP produced by mitochondrial respiration (26). Thus, mitochondrial inhibitors strongly repress hyphal growth by reducing total intracellular levels of ATP. Defects in pyruvate metabolism and the TCA cycle also lead to defective hyphal formation. Conversely, high levels of glucose repress mitochondrial function, leading to reduced filamentation (32, 34). Therefore, both glycolysis and ATP production by the TCA cycle are important drivers of C. albicans filamentation and likely microcolony formation as well.

We sought to define the signaling pathways that control microcolony formation in C. albicans, given the critical role this early morphogenetic response plays in host cell attachment and virulence. We found that the Ras1-cAMP pathway was required for microcolony formation, while the Cek1 pathway was dispensable and the HOG pathway provided negative regulation. We identified a novel function for Sho1 in regulating the Ras1-cAMP pathway, in part because of its ability to regulate the expression of core microcolony genes involved in fungal adhesion and invasion. We further demonstrate that Sho1 integrates the canonical glycolytic pathway to initiate microcolony formation in C. albicans.

## RESULTS

### Glycolysis is a key pathway needed for production of microcolonies in C. albicans.

**FIG 2:**
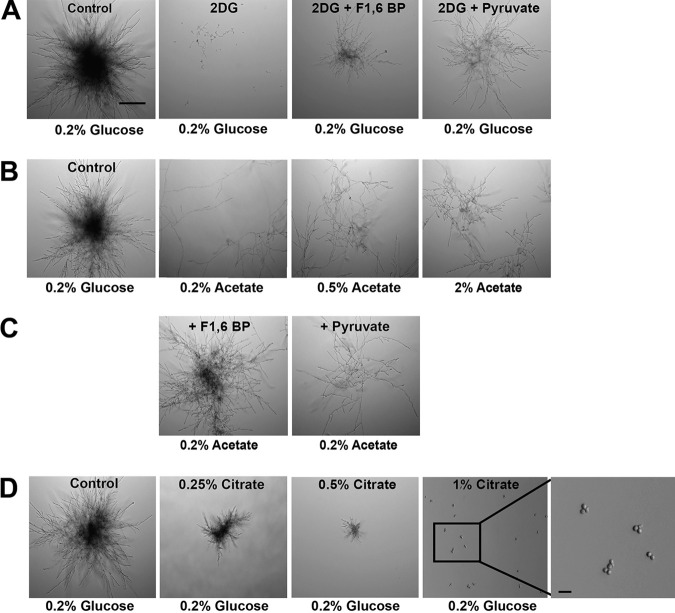
Glycolysis plays a central role in C. albicans microcolony formation. Microcolony formation was assessed in C. albicans SC5314 cultured at 37°C and 5% CO_2_ in RPMI supplemented with the following: 0.1% 2-deoxy-d-glucose (2-DG) in 0.2% glucose with 5 mM fructose 1,6-bisphosphate (F1,6-BP) or 5 mM sodium pyruvate (A); sodium acetate (0.2%, 0.5%, and 2%) (B); 0.2% acetate with 5 mM F1,6-BP or 5 mM sodium pyruvate (C); or 0.2% glucose with sodium citrate (0.25%, 0.5%, and 1%) (D). Cells were not able to form microcolonies in the nonfermentable substrate 2-DG, although addition of both the glycolytic intermediates F1,6-BP and pyruvate restored microcolony formation (A). Acetate was not able to support microcolony formation at concentrations equivalent to that of glucose (0.2%), although small, thin microcolonies were formed at higher acetate concentrations (B). Addition of the glycolytic intermediate F1,6-BP to 0.2% acetate restored microcolony formation; however, addition of the glycolysis end product pyruvate did not restore this phenotype (C). Addition of citrate resulted in a concentration-dependent repression of microcolony formation (D). Bars, 100 μm (A) and 10 μm (D).

To understand the requirements of glycolysis and the TCA cycle for microcolony formation, we tested whether blocking either of these pathways impacted microcolony development. 2-Deoxy-d-glucose (2DG) is a stable glucose analogue that cannot be utilized by yeast in glycolysis (35). Cells were grown in medium with 0.2% glucose, and the addition of 2DG resulted in the repression of microcolony formation (Fig. 2A). Addition of the glycolysis intermediate fructose 1,6-bisphosphate (F1,6-BP) and the glycolysis end product pyruvate (which is converted to acetyl coenzyme A [acetyl-CoA] before entering the TCA cycle) to the same medium resulted in restoration of the phenotype (Fig. 2A). Acetate, a nonfermentable carbon source that is metabolized into acetyl-CoA, which directly enters the TCA cycle (31), was next examined as a substrate for microcolony formation. Acetate was not able to support microcolony formation at concentrations equivalent to that of glucose (0.2%), although 5- to 10-fold-higher acetate concentrations (0.5% to 2%) did result in formation of very small and thin microcolonies (Fig. 2B). Addition of F1,6-BP to 0.2% acetate restored microcolony formation; however, addition of pyruvate did not (Fig. 2C). These results suggested that energy produced from glycolysis is needed for microcolony formation but that the TCA cycle alone is not sufficient to support microcolony formation in C. albicans. To further test this possibility, citrate was added to 0.2% glucose, because citrate specifically represses the production of F1,6-BP by inhibiting the enzyme PFK1 (36). Indeed, we observed a concentration-dependent loss of microcolony formation with the addition of increasing citrate concentrations (0.25%, 0.5%, and 1%), and the pH of the medium was adjusted using 0.5 N NaOH to maintain the optimum physiological pH, ∼7.2, which corresponded to citrate repression (Fig. 2D), further showing the crucial role of glycolysis for microcolony production.

### Microcolony formation in C. albicans is mediated by the cAMP-PKA pathway.

**FIG 3:**
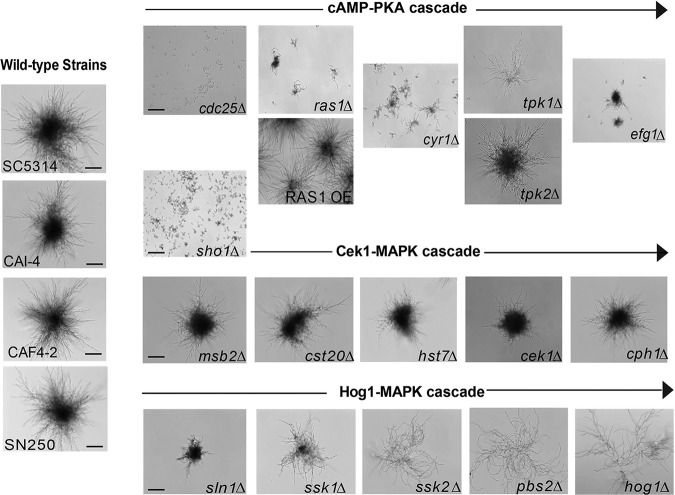
C. albicans microcolony formation requires both Sho1 and Ras1-cAMP pathway components. Microcolony phenotypes of C. albicans parental strains (SC5134, CAI-4, CAF4-2, and SN250) are shown at left. cAMP-PKA mutants, including the *cdc25*Δ, *ras1*Δ, *cyr1*Δ, *tpk1*Δ, and *efg1*Δ strains, all had severe defects in microcolony formation, while *RAS1* overexpression (*RAS1* OE) and *tpk2*Δ deletion strains had phenotypes similar to those of the parental strains. Among the Cek1-MAP kinase pathway mutants, the *sho1*Δ strain was completely devoid of microcolonies, while all other single-gene deletion mutants (*msb2*Δ, *cst20*Δ, *hst7*Δ, *cek1*Δ, and *cph1*Δ strains) had no defects in microcolony formation. The Hog1-MAPK pathway *sln1*Δ and *ssk1*Δ deletion mutants formed smaller but recognizable microcolonies, although the strains with more downstream mutations, i.e., the *ssk2*Δ, *pbs2*Δ, and *hog1*Δ mutants, formed large microcolonies with elongated hyphae. Bars, 100 μm.

To determine which of the three MAPK signaling cascades (Hog1, Cek1, and cAMP-PKA) known to initiate filamentation in response to environmental stimuli might be involved, we examined C. albicans mutants carrying a gene deletion for each component of the cAMP-PKA cascade, the Cek1 pathway, and the Hog1-MAPK pathway (Fig. 3) for their ability to form microcolonies in RPMI (containing 0.2% glucose) medium. C. albicans wild-type (WT) parental strains, including SC5314, CAI-4, CAF4-2, and SN250, all formed typical microcolonies characterized by long filamentous hyphae radiating from a dense center (Fig. 3). We expected that Efg1 deletion mutants in the Ras1-cAMP signaling cascade would demonstrate the most severe deficiency in microcolony phenotype. Indeed, C. albicans *efg1*Δ cells formed very few microcolonies (Fig. 3), since these cells also had limited hypha formation (see Fig. S1 in the supplemental material). Interestingly, the C. albicans *ras1*Δ deletion, as well as deletions of its associated proteins (*cdc25*Δ and *cyr1*Δ), also resulted in severely defective or absent microcolonies (Fig. 3). The importance of Ras1 for microcolony formation was further illustrated by the robust microcolony phenotype shown by the Ras1 overexpression strain (*RAS1* OE). Moreover, among the TPK1 catalytic isoforms of the cAMP-dependent PKA, the *tpk1*Δ mutant formed atypical microcolonies with wispy hyphae without any compact dense center, while deletion of its opposing repressor subunit (*tpk2*Δ) resulted in typical microcolonies (Fig. 3). Although *sho1*Δ, *ras1*Δ, and *cyr1*Δ deletion mutants were significantly defective in microcolony formation, each of these strains was able to germinate and form either hyphae or pseudohyphae in liquid medium with *N*-acetylglucosamine at 37°C and RPMI (Fig. S1 and S2); thus, this defect was specific to microcolony formation rather than a general filamentation defect (unlike hyphal defects exhibited by *efg1*Δ and *cdc25*Δ strains [Fig. S1]).

10.1128/mSphere.00366-20.1 FIG S1 C. albicans strain SC5314 and the *sho1*Δ, *ras1*Δ, *cdc25*Δ, *cyr1*Δ, and *efg1*Δ mutants were grown in *N*-acetyl-d-glucosamine and RPMI 1640 at 37°C for 4 h with continuous shaking at 220 rpm. All mutants except *cdc25*Δ and *efg1*Δ strains were able to filament in the presence of *N*-acetyl-d-glucosamine. In RPMI, the wild-type, *sho1*Δ, *ras1*Δ, and *cyr1*Δ strains formed small filaments or pseudohyphal structures. Both the *cdc25*Δ and *efg1*Δ mutants were unable to filament when cultured in RPMI. Bar, 10 μm. Download FIG S1, EPS file, 1.8 MB. Copyright © 2020 Kumar et al. 2020 Kumar et al. This content is distributed under the terms of the Creative Commons Attribution 4.0 International license.

10.1128/mSphere.00366-20.2 FIG S2 (A) For growth curve analysis, C. albicans SC5314 and the *sho1*Δ strain were cultured for 12 h at 37°C and then diluted to an OD_600_ of 0.1 in RPMI alone or RPMI supplemented with 0.1% 2-deoxyglucose (2-DG), 0.2% sodium acetate, and 0.2% sodium acetate with 5 mM fructose 1,6-bisphosphate (F1,6-BP). Cells were then cultured at 37°C with continuous shaking at 220 rpm for 24 h, and OD_600_ was measured at various time points. There were no significance differences in growth curves between the *sho1*Δ and WT strains among the culture media. (B) Percent germination of C. albicans SC5314 and *sho1*Δ strains was calculated as the number of germinated cells relative to total observed C. albicans cells. C. albicans strains were grown for 12 h in YPD, diluted to an OD_600_ of 0.3 to 0.4 in RPMI or YNB +1.25% *N*-acetyl-d-glucosamine (GlcNAc; Sigma), and incubated for 2 h to 4 h at 37°C to induce germination. Both RPMI and YNB+GlcNAc were supplemented with 0.1% 2-DG, 0.2% sodium acetate, or 0.2% sodium acetate with 5 mM F1,6-BP. Images were acquired using bright-field microscopy, and hyphal formation was determined in multiple fields from at least 100 cells. Download FIG S2, EPS file, 0.7 MB. Copyright © 2020 Kumar et al. 2020 Kumar et al. This content is distributed under the terms of the Creative Commons Attribution 4.0 International license.

Next, we examined the Cek1-MAPK pathway, which is known to activate cell wall remodeling in conjunction with morphogenesis. Surprisingly, none of the deletion mutants with deletions in the Cek1-MAPK pathway showed a deficit in microcolony phenotype, as C. albicans *msb2*Δ, *cst20*Δ, *hst7*Δ, *cek1*Δ, and *cph1*Δ mutants all formed microcolonies similar to those of the WT (Fig. 3). But unexpectedly, deletion of the membrane adaptor protein Sho1, which regulates downstream kinases presumably in the Cek1 pathway, resulted in total loss of microcolony formation. Although Sho1 is believed to link stress responses to Cek1-mediated morphogenesis and cell wall biosynthesis, this result suggests that Sho1 has a function in microcolony formation that is independent of the Cek1 cascade in C. albicans and that may be coupled with the Ras1-cAMP pathway.

Last, we examined strains with gene deletions in the Hog1-MAP kinase pathway. Deletion of the putative head sensor Sln1 had a small defect in microcolony formation similar to the mild defect observed in *ssk1*Δ cells (Fig. 3). Interestingly, cells carrying deletions of the more downstream elements, including C. albicans *ssk2*Δ, *pbs2*Δ, and *hog1*Δ mutants, showed a distinctive phenotype consisting of highly dispersed elongated hyphae with no dense center (Fig. 3). These Hog1-MAP kinase mutants formed less organized but extensive hyphal structures instead of the more densely packed microcolony phenotype of parental strains. Thus, the HOG pathway may function to oppose microcolony formation.

### Sho1 mediates microcolony formation as an upstream element of the Ras1-cAMP pathway.

**FIG 4:**
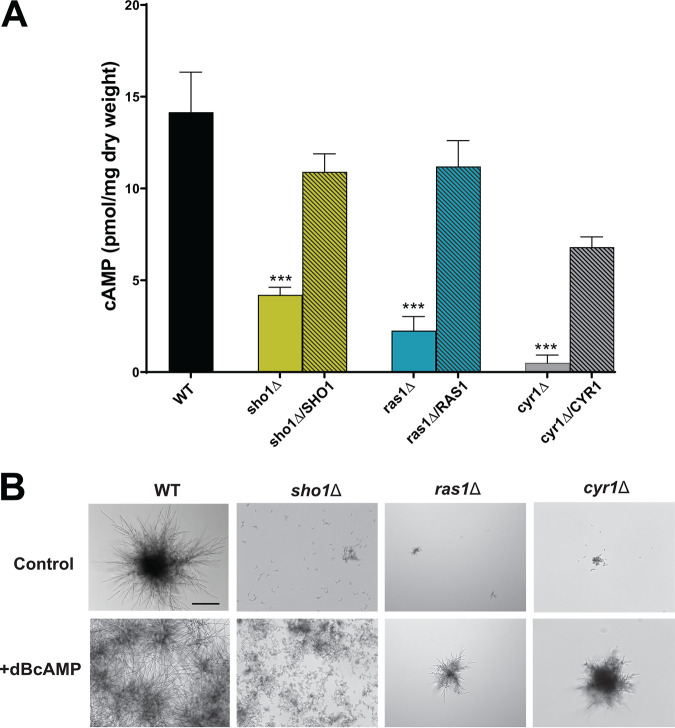
Exogenous addition of cAMP restores microcolony formation to C. albicans *ras1*Δ but not *sho1*Δ mutant strains. (A) Intracellular cAMP was collected from cell lysates of C. albicans microcolonies (wild type [WT] and mutants) cultured in RPMI at 37°C in 5% CO_2_. cAMP concentrations were determined by comparison to a standard curve, and significant differences were determined by one-way ANOVA. cAMP levels were significantly (***, *P* < 0.001) reduced in *sho1*Δ, *ras1*Δ, and *cyr1*Δ mutants compared to the WT, while cAMP levels were not different from that of the WT in complemented strains. (B) Addition of exogenous cAMP (100 mM) to WT strains increased microcolony density, while cAMP addition to *sho1*Δ mutants increased hyphal formation but did not produce organized microcolonies. However, addition of cAMP to *ras1*Δ and *cyr1*Δ mutants restored a microcolony phenotype, although microcolonies remained smaller than those of WT cells. Bars, 100 μm.

The major role of the Ras1-cAMP is the synthesis and accumulation of intracellular cAMP (15, 24), which is essential for many cellular processes, including filamentation and microcolony formation. Given that the *sho1*Δ mutant phenocopied the *ras1*Δ mutant in its defect in microcolony formation, we investigated whether Sho1 might share other Ras1-dependent functions. To define whether and how Sho1 might connect to the Ras1-cAMP pathway, we first asked if Sho1 is needed for cAMP production. Intracellular cAMP levels in microcolonies of *sho1*Δ cells were significantly (*P* < 0.001) reduced 3-fold compared to WT cells, while the complemented strain *sho1*Δ/SHO1 produced cAMP nearly at WT levels (Fig. 4A). C. albicans *ras1*Δ and *cyr1*Δ cells had severe defects in cAMP production (4- to 9-fold), as might be expected due to the direct role of these genes in cAMP production. This result suggests that there is a regulatory link between Sho1 and the Ras1-cAMP pathway. To further assess this possibility, we tested whether addition of cAMP in *sho1*Δ, *ras1*Δ, and *cyr1*Δ deletion mutants would functionally complement microcolony defects. Interestingly, addition of cAMP to WT cells increased the length of hyphae and numbers of WT microcolonies (Fig. 4B) and complemented the loss-of-microcolony phenotype of *ras1*Δ and *cyr1*Δ cells. In contrast, addition of cAMP to *sho1*Δ cells resulted in a denser aggregate of cells but did not restore microcolony formation. Thus, while Sho1 is needed for cAMP production as part of the Ras1-cAMP pathway, its function is not restored by cAMP, suggesting that it functions upstream of the Ras1-Cyr1 complex, which generates cAMP in the Ras1-cAMP pathway.

### C. albicans mutants with deletions of *SHO1* or *RAS1* are unable to induce transcription of core microcolony genes.

**FIG 5:**
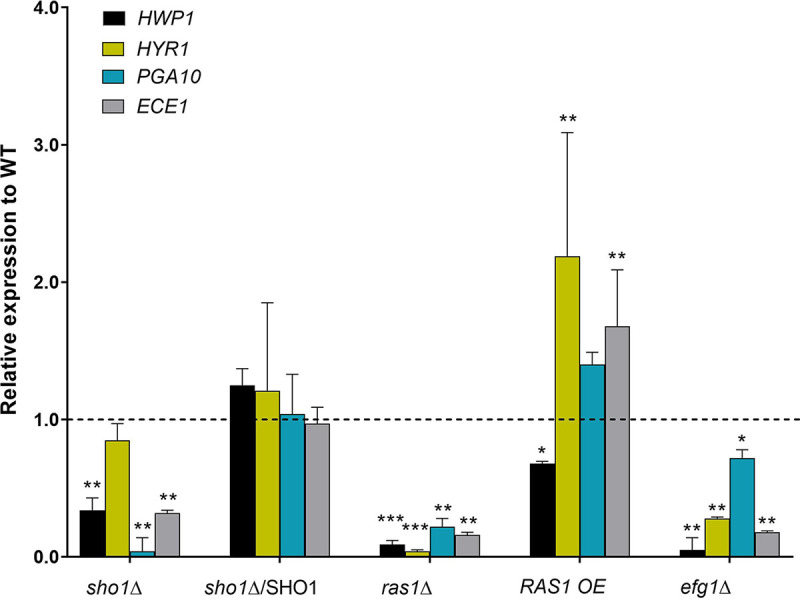
Quantitative real-time RT-PCR analysis of core genes expressed during microcolony formation in C. albicans. Expression of the core microcolony genes *HWP1*, *HYR1*, *PGA10*, and *ECE1* was determined by RT-qPCR from microcolonies of C. albicans *sho1*Δ, *ras1*Δ, *efg1*Δ, and *RAS1* OE strains and compared to that of the parental wild type grown in RPMI at 37°C in the presence of 5% CO_2_. Significantly reduced expression of core microcolony genes was found in *sho1*Δ, *ras1*Δ, and *efg1*Δ mutants compared to the wild type. However, the expression of these genes was restored in the complemented *sho1*Δ/*sho1*Δ::SHO1 strain and significantly increased in the *RAS1* OE strain. Gene expression levels are shown as relative fold changes in RNA expression compared to the wild type from the corresponding standard curves using *ACT1* as a control. Data are means and standard errors of the means (SEM) for three replicates from at least two independent experiments. *P* values for comparisons between control and experimental groups were determined by one-way ANOVA: *, *P < *0.05; **, *P < *0.01; ***, *P < *0.001.

To further assess the role of Sho1 in microcolony formation, we measured expression levels of four core microcolony genes in C. albicans *sho1*Δ and *ras1Δ* cells. Among genes most highly expressed specifically with microcolony formation (5), we selected *HWP1*, *HYR1*, *ECE1*, and *PGA10* as representative genes to analyze using reverse transcription-quantitative PCR (RT-qPCR) in *sho1*Δ, *sho1*Δ/*SHO1*, *ras1*Δ, *RAS1* OE, and *efg1*Δ strains. When normalized to those of WT cells, the expression levels of three genes were significantly reduced (0.3- to 0.01-fold) in *sho1*Δ cells, and these levels were restored to those of WT cells in the complemented *sho1*Δ/*SHO1* strain (Fig. 5). C. albicans *ras1*Δ cells had significantly reduced expression of all four genes, and the overexpression strain *RAS1* OE had significantly increased expression of three of these genes. As a control, cells with deletion of *EFG1*, which encodes the most downstream component of the Ras1-cAMP pathway, also had significantly reduced expression of all four microcolony genes (Fig. 5). Thus, Sho1 and Ras1 are both essential elements for expression of core microcolony genes, which supports the idea that both proteins are components of the Ras1-cAMP pathway mediating microcolony formation.

### Sho1 mediates signaling between the glycolytic pathway and microcolony formation in C. albicans.

S. cerevisiae Sho1 is an adaptor protein that interacts with multiple partners, including proteins that make up the glycolysis pathway (37). A search for potential interactions between S. cerevisiae glycolytic proteins and signaling pathways using the S. cerevisiae interactome BIOGRID database also identified members of the Ras1-cAMP pathway (Table 1). Thus, in S. cerevisiae, there may be physical interactions between Sho1 and glycolytic enzymes that also regulate the Ras1-cAMP pathway and its target genes. Based upon this information, the *Candida* genome database (CGD) was used to predict C. albicans orthologs for S. cerevisiae genes. Four interacting C. albicans glycolytic enzymes (Pfk1, Fba1, Pgk1, and Cdc19) were predicted to interact with SH3 or transmembrane (TM) domains of C. albicans Sho1 (Table 1). Furthermore, these glycolytic enzymes are highly homologous to S. cerevisiae gene products predicted to have physical interactions with the Ras GTPase-activating protein (GAP) encoded by *IRA2* and/or *TPK1*/*TPK2* in the Ras1-cAMP pathway (Table 1).

**TABLE 1:** Predictions for C. albicans Sho1 protein-protein interactions^*a*^

S. cerevisiae Sho1p-interacting partner		Predicted C. albicans Sho1p-interacting ortholog (glycolysis)	Interacting Sho1p domain
Glycolysis	Ras1-cAMP		
Fba1	*IRA2*	Fba1	SH3
Pfk1	*TPK1*	Pfk1	TM
Pgk1	*IRA2*	Pg1	TM
Cdc19	*TPK1*/*TPK2*	Cdc19	TM

a Fba1, fructose-bisphosphate aldolase; Pfk1, phosphofructose kinase; Pg1, phosphoglycerate kinase; Cdc19, pyruvate kinase.

**FIG 6:**
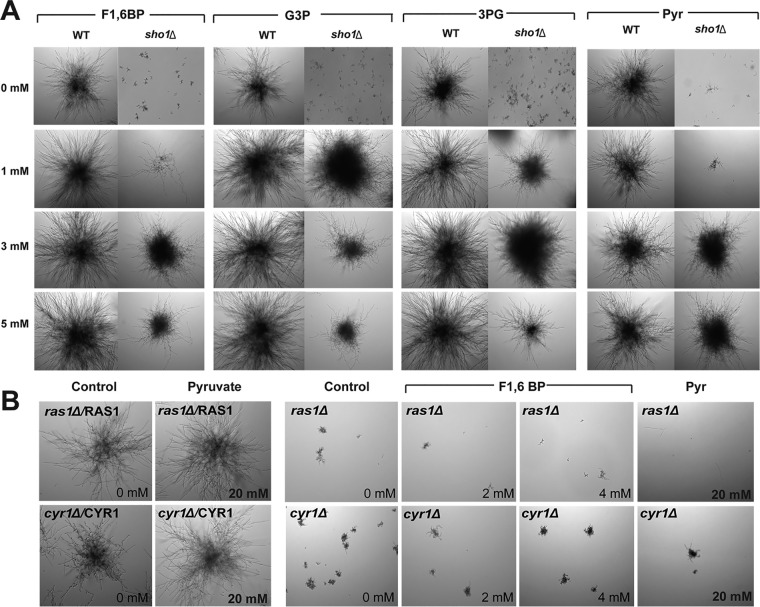
Intermediates of the glycolytic pathway bypass the requirement for Sho1 during microcolony formation in C. albicans. (A) Wild-type C. albicans strain SC5314 and the *sho1*Δ mutant were cultured at 37°C in the presence of 5% CO_2_ and RPMI supplemented with 1 mM, 3 mM, or 5 mM fructose 1,6-bisphopshate (F1,6-BP), glyceraldehyde-3-phosphate (G3P), 3-phosphoglycerate (3-PG), and pyruvate (Pyr). A microcolony phenotype was restored in *sho1*Δ cells following the addition of F1,6-BP (3 and 5 mM), G3P (1, 3, and 5 mM), 3-PG (1, 3, and 5 mM), and Pyr (3 and 5 mM). (B) The ras*1*Δ, cyr*1*Δ, and respective complemented strains (*ras1*Δ/*RAS1* and *cyr1*Δ/*CYR1* strains) were cultured at 37°C in the presence of 5% CO_2_ and RPMI supplemented with 0 and 4 mM concentrations of F1,6-BP and 0 and 20 mM pyruvate (Pyr). The defective microcolony phenotype of *ras1*Δ and *cyr1*Δ was not restored in the presence of F1,6-BP and pyruvate.

We predicted that if C. albicans Sho1 interacts with and regulates these glycolytic enzymes, addition of intermediates of glycolysis might restore microcolony formation in the *sho1*Δ mutant. To test this hypothesis, microcolony formation was examined in *sho1*Δ cells after addition of fructose 1,6-bisphosphate (F1,6-BP) to bypass Pfk1 function, glyceraldehyde 3-phosphate (G3P) to bypass Fba1 function, 3-phosphoglycerate (3PG) to bypass Pgk1 function, and pyruvate (Pyr) to bypass Cdc19 function (Fig. 6). F1,6-BP was able to partially restore the microcolony phenotype at higher concentrations (3 mM and above). In contrast, addition of 1 mM G3P resulted in restoration of the microcolony phenotype. At high concentrations of G3P (3 to 5 mM), a reduced microcolony phenotype was observed, which might indicate feedback repression. 3PG also restored a microcolony phenotype in the *sho1*Δ mutant so that 3 mM 3PG produced even larger microcolonies than WT cells, while addition of 5 mM 3PG repressed this phenotype. Addition of Pyr to *sho1*Δ cells also restored microcolony formation but only at higher concentrations (3 to 5 mM) and without evidence of feedback repression. This effect was specific to Sho1, because *ras1*Δ and *cyr1*Δ deletion mutants showed no impact on microcolony phenotype with the addition of F1,6-BP or Pyr (Fig. 6B). These results support *in silico* predictions that Sho1 interacts with glycolytic enzymes as part of a regulatory mechanism linking glycolysis with the Ras1-cAMP pathway.

## DISCUSSION

Microcolony formation in C. albicans represents a unique adaptation of this virulent fungal pathogen for colonization of host tissues. This distinctive feature is governed by Sfl1/Sfl2 and Efg1 transcriptional regulators (4). These transcription factors are central in microcolony formation through regulation of a distinct set of target genes (4). Until now, signaling pathways linking environmental cues, particularly carbon utilization, to transcriptional responses needed for microcolony formation have been undercharacterized. Among signaling pathways, a role for Ras1-cAMP in regulating Sfl2- and Efg1-mediated transcription for wrinkled colony (microcolony) growth was identified in response to CO_2_ (9), and the tricarboxylic acid (TCA) cycle has been shown to impact ATP and cAMP levels to regulate Ras1-cAMP signaling (9, 26, 32).

Here, we describe a new regulatory connection between Sho1 and the Ras1-cAMP pathway. Little is known about the function of Sho1 in C. albicans, although it has been implicated in diverse roles in morphogenesis and cell wall biosynthesis (14, 17, 38), in modulating activity of the antifungal peptide Hst 5 (39), and as a virulence factor (40). Sho1 is known to have a role in filamentation on solid surfaces, and its deletion resulted in defective agar invasion (14), similar to what we found for microcolony formation. Here, we establish that Sho1 is necessary for transcription of core microcolony virulence genes that are associated with hyphal formation (*HWP1*, *HYR1*, and *ECE1*) and nutrient acquisition (*PGA10*) as well as maintaining intracellular cAMP levels necessary for Ras1-cAMP signaling.

**FIG 7:**
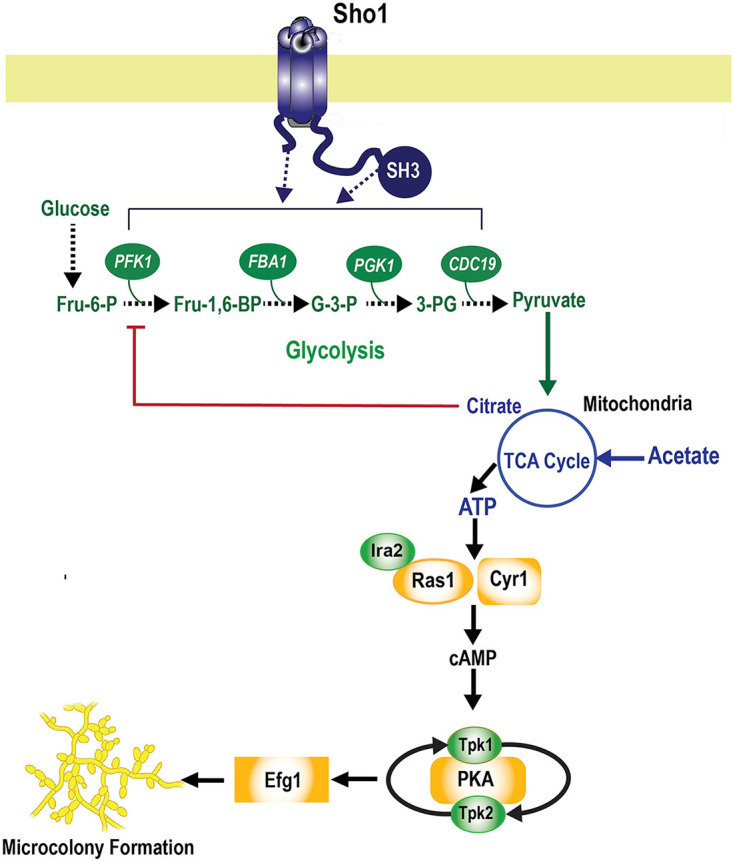
Model of Sho1 glycolysis-mediated signaling through glycolysis and Ras1-cAMP in microcolony formation. The model places Sho1, a transmembrane adaptor protein, as the most upstream element of the signaling pathway that interacts with enzymes of the glycolysis pathway and TCA cycle before the signal is translated to downstream targets such as Ras1 and Cyr1 and C. albicans microcolonies are formed. Either the SH3 domain of the Sho1 protein interacts with phosphofructose kinase 1 (Pfk1) or its TM domain interacts with fructose-bisphosphate aldolase 1 (Fba1), phosphoglycerate kinase 1 (Pgk1), and pyruvate kinase (CDC19) in mediating the signaling response.

S. cerevisiae Sho1 is a membrane-bound adaptor protein composed of four transmembrane (TM) domains and cytoplasmic Src homology 3 (SH3) domain at its C terminus. S. cerevisiae Sho1 forms planar oligomers that allow it to assume a scaffold structure that is able to bind with multiple other proteins, including Opy2/Hkr1 (a subbranch of Hog1) and Msb2/Opy2 (a subbranch of Hog1), which results in Hog1 (41–43) and Cek1 (44) signaling. The S. cerevisiae TM domain of Sho1 also interacts with selected glycolytic enzymes (Pfk1, Fba1, Pgk1, and Cdc19) (37), and we identified further physical interactions with Ras1-cAMP genes (*TPK1*, *TPK2*, and *IRA2*), suggesting signaling through this pathway. Structurally, C. albicans Sho1 also contains four TMs and an SH3 domain (Fig. 7) that we predicted to be involved in similar protein–protein interactions in the glycolytic pathway (Table 1). To provide a functional connection between Sho1 and glycolysis with microcolony formation in C. albicans, we tested whether replacement of carbon substrates downstream of predicted interaction sites would restore microcolony formation in C. albicans *sho1*Δ deletion mutants. Interestingly, addition of any of the carbon substrates was able to restore microcolony formation and bypass Sho1, suggesting that redundant mechanisms may substitute for Sho1 interactions with sufficiently high concentrations of some carbohydrate intermediates. For example, microcolony restoration required supplementation of at least 3 mM F1,6-BP (the enzymatic product of Pfk1) or pyruvate (the enzymatic product of *cdc19*), while only a 1 mM concentration of other intermediates was needed to produce microcolonies in a *sho1*Δ deletion mutant. Since both enzymes catalyze glycolysis-committed steps, the absence of Sho1 may alter the kinetics so that a much higher level of substrate is required to drive catalysis (33). We also noted that addition of G3P and 3PG substrates caused repression of the microcolony size phenotype at higher concentrations, suggesting the occurrence of some form of catabolite repression that may reduce the transcription of genes controlling functions such as filamentation (32, 45). The role of Sho1 as a functional partner for these glycolytic enzymes was further supported in that addition of the same glycolytic intermediates to *ras1*Δ or *cyr1*Δ deletion mutants did not restore any microcolony phenotype (Fig. 6B).

Although we did not further assess the role of C. albicans Sho1 in the Hog1 MAPK pathway, there is likely some regulatory role, based on the aberrant microcolony structures observed in deletion mutants of the Hog1 pathway (Fig. 3) as well as the predicted interaction between S. cerevisiae Sho1 and Pbs2 (the kinase activated by osmotic and oxidative stress-regulating Hog1) (34). One previous study found reversal of loss of pseudohyphal growth of C. albicans *sho1*Δ deletion mutants on solid media upon additional deletion of *HOG1*, suggesting a repressive role for Hog1 (14). This is consistent with our finding that *pbs2*Δ and *hog1*Δ cells had highly elongated filaments in their microcolony phenotype, which was not as evident in mutants with deletions in more upstream Hog1 pathway members (Fig. 3), also suggesting a repressive role for Pbs2 and Hog1. Additionally, S. cerevisiae Sho1 also shares a common SH3 domain with Cdc25, a Ras1 effector that binds adenylyl cyclase and facilitates Ras1-mediated cAMP signaling (46). Interestingly, in this study, the C. albicans *cdc25*Δ deletion mutant showed a yeast-locked phenotype and failed to undergo filamentation even in liquid inducing medium (*N*-acetyl-d-glucosamine) (Fig. S1). It is possible that C. albicans Sho1 may also regulate Cdc25 through binding with its SH3 domain to alter filamentation. Further studies to validate physical binding interactions between Sho1 and these predicted partners are needed.

Our results define a new role for C. albicans Sho1 in signaling via the Ras1-cAMP pathway. Future studies exploring the functional relevance of protein interactions linking Sho1 to glycolysis may further our understanding of C. albicans microcolonies as a form of infection in the human host.

## MATERIALS AND METHODS

### Strains and media.

The C. albicans strains used and their genotypes are listed in Table 2. C. albicans SC5314 was kindly provided by David Roberts (National Institutes of Health, Bethesda, MD), CAI-4 and the *cek1*Δ strain were kindly provided by Malcolm Whiteway (Concordia University, Montreal, Canada), the *hog1*Δ strain was kindly provided by Janet Quinn (Newcastle University, Newcastle, United Kingdom), and the *cyr1*Δ, *tpk1*Δ, *tpk2*Δ, *efg1*Δ, *ras1*Δ, *cyr1*Δ/*CYR1*, *ras1*Δ/*RAS1*, and *ras1*-G13V (*RAS1* OE) strains were provided by Deborah Hogan (Dartmouth College). All C. albicans strains were cultured for 12 h in yeast extract-peptone-dextrose medium (YPD; BD Difco) broth supplemented with 50 μg/ml of uridine (Sigma) at 30°C in an orbital shaker at 220 rpm. C. albicans cells were pelleted by centrifugation at 2,500 × *g* for 5 min, washed three times in phosphate-buffered saline (PBS; pH 7.4; Thermo Fisher Scientific) and resuspended in RPMI 1640 supplemented with l-glutamine (Corning Cellgro) for experiments.

**TABLE 2:** C. albicans strains used in this study

Strain or mutation	Genotype	Reference
SC5314	Prototrophic clinical isolate	48
CAI-4	Δ*ura3*::*imm434*/*URA3*	48
CAF4-2	*URA3*/*ura3*::λ*imm434 IRO1*/*iro1*::λ*imm434*	48
SN250	*ura3*::*imm434*::*URA3*/*ura3*::*imm434 iro1*::*IRO1*/*iro1*::*imm434 his1*::*hisG*/*his1*::*hisG leu2*/*leu2 arg4*/*arg4*	49
*cdc*25Δ	Δ*ura3*::λ*imm434*/*ura3*::λ*imm434 arg4*::*hisG*/*arg4*::*hisG his1*::*hisG*/*his1*::*hisG* Δ*cdc25*::*HIS1*/*cdc25*::*ARG4*	26
*ras1*/*ras1*-*G13V* (*RAS1* OE)	Δ*ura3*::λ*imm434*/Δ*ura3*::λ*imm434 ras1*::*hisG*/*ras1*::*hisG*::*ras1-G13V-URA3*	50
*ras1*Δ	Δ*ura3*::λ*imm434*/Δ*ura3*::λ*imm434*Δ*ras1*::*hisG*/*ras1*::*hisG*::*URA3*	50
*cyr1*Δ	Δ*ura3*::*kimm434*/Δ*ura3*::*kimm434*Δ*cyr1*::*hisG*::Δ*cyr1*::*hisG*	51
*ras1*Δ/*RAS1*	Δ*ura3*::λ*imm434*/Δ*ura3*::λ*imm434*Δ*ras1*::*hisG*/Δ*ras1*::*hisG*::*RAS1-URA3*	50
*cyr1*Δ/*CYR1*	Δ*ura3*::λ*imm434*/Δ*ura3*::λ*imm434*Δ*cyr1*::*hisG*/Δ*cyr1*::*hisG*::*CYR1-URA3*	50
*tpk1*Δ	Δ*ura3*::λ*imm434*/Δ*ura3*::λ*imm434 his1*::*hisG*/*his1*::*hisGarg4*::*hisG*/*arg4*::*hisG tpk1*::*ARG4*/*tpk1*::*URA3*	50
*tpk2*Δ	Δ*ura3*::λ*imm434*/Δ*ura3*::λ*imm434 his1*::*hisG*/*his1*::*hisGarg4*::*hisG*/*arg4*::*hisG tpk2*::*ARG4*/*tpk2*::*URA3*	50
*efg1*Δ	Δ*ura3*::λ*imm434*/Δ*ura3*::λ*imm434efg1*::*hisG*/*efg1*::*hisA*-*hisG*-*ura3*::λ*imm434*/*ura3*::λ*imm434efg1*::*hiG*	52
*sho1*Δ	Δ*ura3*::*imm434*/Δ*ura3*::*imm434* Δ/Δ*sho1*::*URA*	Edgerton lab
*sho1*Δ/SHO1	Δ*ura3*::*imm434*/Δ*ura3*::*imm434* Δ*sho1*::*URA*/*SHO1*	Edgerton lab
*msb2*Δ	Δ*ura3*::*imm434*/Δ*ura3*::*imm434* Δ/Δ*msb2*::*URA*	22
*cst20*Δ	Aaron Mitchell kinase collection	Mitchell lab
*hst7*Δ	Aaron Mitchell kinase collection	Mitchell lab
*cek1*Δ	Δ*ura3*::*imm434*/Δ*ura3*::*imm434* Δ*cek1*::*hisG-URA-hisG*/Δ*cek1*::*hisG*	19
*cph1*Δ	Δ*cph1*:*hisG*/Δ*cph1*::*hisG-URA3-hisG* Δ*ura3*/Δ*ura3*	48
*sln1*Δ	Δ*ura3*::*imm434* Δ*sln1*::*FRT*/Δ*ura3*::*imm434*Δ*sln1*::*FRT*	Edgerton lab
*ssk1*Δ	Δ*ura3*::*imm434*/Δ*ura3*::*imm434* Δ*ssk1*::*hisG*/Δ*ssk1*::*hisG-URA3-hisG*	15
*ssk2*Δ	Δ*ura3*::*imm434*/Δ*ura3*::*imm434* Δ*ssk2*::*hisG*/Δ*ssk2*::*hisG-URA3-hisG*	15
*pbs2*Δ	Δ*pbs2*::*hisG*/Δ*pbs2*::*hisG-URA3-hisG* Δ*ura3*/Δ*ura3*	53
*hog1*Δ	Δ*ura3*::*imm434*/Δ*ura3*::*imm434 his1*::*hisG*/*his1*::*hisG hog1*::*loxP-ura3-loxP*/*hog1*::*loxP-HIS1-loxP CIp20*	54

### Microcolony formation.

C. albicans strains were grown for 12 h in YPD at 30°C in an orbital shaker at 220 rpm, washed twice with PBS, and diluted to 10^3^ cells/ml in PBS. A total of 100 C. albicans cells were added to 1 ml RPMI medium (Corning Cellgro with 0.2 g/liter glucose, as in the original formulation) or RPMI no-glucose (RPMI NG) medium (Sigma R1383) supplemented with 0.2% glucose or other glycolysis intermediates per well of a 12-well cell culture plate (no. 353225; BD Falcon) and incubated at 37°C for 16 h in 5% CO_2_. For cAMP experiments, 3′,5′-cyclic AMP sodium salt (Sigma) was added to RPMI medium (final concentration of 100 mM) before addition to yeast cells. For the assessment of glycolytic pathway components, fructose 1,6-bisphosphate (Sigma), 3-phosphoglyceric acid (Sigma), glyceraldehyde 3-phosphate (Sigma), or sodium pyruvate (Sigma) was added to RPMI medium, and C. albicans cells were tested for the formation of microcolonies. To assess the effect of alternate carbon sources on microcolony production, cells were incubated in RPMI NG supplemented with sodium acetate (0.2%, 0.5%, or 2%) (Sigma) as the carbon source, RPMI supplemented with sodium citrate (0.25%, 0.5%, or 1%), or RPMI supplemented with 0.1% 2-deoxy-d-glucose (Sigma) and subsequently imaged. The optimum physiological pH was maintained using 0.5 N NaOH. Microcolonies were observed using bright-field microscopy with a Zeiss Axio Observer Z1 microscope at a magnification of ×10.

### Growth assays.

C. albicans strains (wild-type SC5314 and *sho1*Δ) were cultured for 12 h in YPD at 30°C in an orbital shaker at 220 rpm and washed twice with PBS. The culture was then diluted to an optical density at 600 nm (OD_600_) of 0.1 in RPMI medium alone or with the addition of 0.1% 2-deoxyglucose (2-DG), 0.2% sodium acetate, or 0.2% sodium acetate with 5 mM F1,6-BP and incubated at 37°C in air with continuous shaking at 220 rpm. Growth of each strain was determined by measuring the OD_600_ at multiple time points.

### Germination assay.

To induce hypha formation, C. albicans strains were grown for 12 h in YPD, diluted to an OD_600_ of 0.3 to 0.4 in RPMI or yeast nitrogen base (YNB) containing 1.25% *N*-acetyl-d-glucosamine (GlcNAc; Sigma), and incubated for 2 to 4 h at 37°C. Both RPMI and YNB+GlcNAc were supplemented with 0.1% 2-deoxyglucose (2-DG), 0.2% sodium acetate, or 0.2% sodium acetate with 5 mM F1,6-BP. Images were acquired using bright-field microscopy with a Zeiss Axio Observer Z1 microscope at a magnification of ×10. Hyphal formation was determined in multiple fields from at least 100 cells, and percent germination was calculated as the number of germinated cells relative to total observed C. albicans cells.

### RNA isolation.

C. albicans cells were inoculated in RPMI 1640 medium in 12-well cell culture plates (200 cells/well) and grown at 37°C for 16 h in 5% CO_2_. The medium was discarded, and 1 ml of 1× PBS was added to the wells. Cells were recovered from each well and pelleted by centrifugation at 2,300 × *g* for 5 min. The cell pellet was resuspended in 1 ml of TRIzol and vortexed (4 cycles, 6 m/s) with 0.45-μm glass beads using a FastPrep-24 5G homogenizer (MP Biomedicals, Santa Ana, CA). After vortexing, 200 μl of chloroform was added to the lysed cells, and the cells were mixed vigorously for 15 s and kept for 2 min at room temperature. Lysed cells were centrifuged at 21,000 × *g* for 10 min at 4°C to separate the upper clear aqueous layer and mixed with 0.5 volume of 100% ethanol to precipitate total RNA from C. albicans cells. Total RNA was further purified using an RNeasy minikit (Qiagen) according to the manufacturer’s instructions. The RNA samples were stored at −80°C.

### SYBR green-based quantitative RT-PCR.

Total RNA from wild-type parental C. albicans strains and the *ras1*Δ, *RAS1* OE, *sho1*Δ, *sho1*Δ/*SHO1*, and *efg1*Δ strains was isolated from microcolonies as described above to quantitate gene expression of selected core microcolony genes (*HWP1*, *HYR1*, *ECE1*, and *PGA10*). Total cDNA was synthesized for each sample using an iScript cDNA synthesis kit (Bio-Rad) following the manufacturer’s instructions, with equal amounts of RNA (1 μg in a 20-μl reaction mixture). Gene expression was quantified by real-time PCR using the iQ SYBR green Supermix (Bio-Rad),150 nM concentrations of both forward and reverse primers, and 50 ng of cDNA template in a total reaction mixture volume of 20 μl. After an initial denaturing step of 3 min at 95°C, the reactions were cycled 40 times with a CFX-96 Touch real-time PCR system (Bio-Rad) under the following conditions: 95°C for 30 s, 55°C for 30 s, and 72°C for 30 s. Each qRT-PCR assay included two negative controls, no template and RNA alone (10 ng), and a positive control, genomic DNA (10 ng). Fluorescent data were collected and analyzed with iCycler iQ software. Relative fold changes in gene expression of *HWP1*, *HYR1*, *ECE1*, and *PGA10* were calculated from the corresponding standard curves compared to *ACT1* as the control. Data shown are the means and standard errors of the means (SEM) for three replicates from at least two independent experiments. Significant differences were determined by one-way analysis of variance (ANOVA).

The primers used for the study are *ACT1*qRT (5′-ACTGCTTTGGCTCCATCTTCT, 3′-TGGATGGACCAGATTCGTCG), *ECE1*qRT (5′-TTGCTAATGCCGTCGTCAGA, 3′-CCAGGACGCCATCAAAAACG), *HWP1*qRT (5′-CTCCTGCCACTGAACCTTCC, 3′-GAGCCAGCTGGAGCAGTTT), *HYR1*qRT (5′-GCTGCTGCCCTTCCACAATA, 3′-GGTGCAGATGGTCCATTGGT), and *PGA10*qRT (5′-GGTGTCGGGGAACCATACTG, 3′-GGAGGTAGTGGCAAGCTCAG).

### Extraction and quantification of intracellular cAMP levels.

Intracellular cyclic AMP (cAMP) was measured as previously described (47) with the following modifications. Microcolonies were grown as described above, and microcolony pellets from each well were transferred to 2-ml FastPrep tubes (MP Biomedicals) containing 100 mg prechilled acid-washed glass beads and 500 μl 10% trichloroacetic acid. Cells were disrupted by 10 cycles of vortexing for 40 s using FastPrep-24 (MP Biomedicals). Samples were placed on ice for 5 min between cycles. After removal of beads, lysates were centrifuged at 4°C for 20 min at 21,000 × *g*. Supernatants were washed five times with water-saturated ether to remove trichloroacetic acid. After trichloroacetic acid neutralization, the supernatant was dried using a Speed Vac in 100 μl of enzyme-linked immunosorbent assay (ELISA) buffer. cAMP was quantified using a cyclic AMP Select ELISA kit (Cayman Chemical) according to the manufacturer’s protocol. In each experiment, a standard curve was generated and used to calculate cAMP concentrations. Experiments were repeated three independent times, and significant differences were determined by one-way ANOVA.

### *In silico* prediction of yeast Sho1-interacting partners.

Interacting proteins for C. albicans Sho1 were predicted based upon the published S. cerevisiae Sho1 protein membrane interactome (37). Glycolytic pathway genes interacting with S. cerevisiae Sho1 TM or SH3 domains were identified using membrane interactome predictions with the *Saccharomyces* Genome Database (SGD; https://www.yeastgenome.org/); then, the BIOGRID interactome database was applied to these genes to identify any MAPK pathway genes interacting physically or genetically with both S. cerevisiae Sho1 and glycolytic genes. The *Candida* Genome Database (CGD; http://www.candidagenome.org) was used to predict C. albicans orthologs for S. cerevisiae genes identified in this screen.
